# Radiographic Appearance of the Urinary Bladder and Application of a Vertebral Bladder Score for Evaluating Bladder Size in Healthy Guinea Pigs (*Cavia porcellus*) and Guinea Pigs with Clinical Signs of Cystitis

**DOI:** 10.3390/ani16060945

**Published:** 2026-03-18

**Authors:** Anika Mische, Kerstin Müller

**Affiliations:** 1 Small Animal Clinic, Freie Universität Berlin, Oertzenweg 19b, 14163 Berlin, Germany; 2 AniCura Veterinary Clinic Haar, Keferloher Str. 25, 85540 Haar, Germany

**Keywords:** caviomorpha, bladder disease, diagnostic imaging, small mammal

## Abstract

The radiographic appearance of the urinary bladder in guinea pigs (*Cavia porcellus*) affected by cystitis is poorly characterized. This retrospective case–control study assessed the radiographic characteristics of the urinary bladder (digital abdominal and whole-body radiographs) in 24 healthy guinea pigs and 24 guinea pigs diagnosed with cystitis. Inclusion criteria for the cystitis cohort included presence of clinical signs (hematuria, incontinence, stranguria, abdominal discomfort, apathy, and inappetence), leukocyturia and/or bacteriuria, complemented by right lateral radiographs and either ultrasound or a positive urine culture. Radiographic criteria indicative of cystitis were systematically evaluated using a vertebral bladder score (VBS) expressed in lumbar vertebral units (LVU) and reference marker lines on lateral radiographs to assess urinary bladder distension. Compared to the control group, radiographs of guinea pigs with cystitis demonstrated increased bladder opacity (46% of cases) and solitary uroliths (25%). Bladder height in patients with cystitis exceeded that of healthy counterparts (median: 16.6 vs. 13.0 mm). The VBS in the cystitis group was significantly elevated (median: 1.50 vs. 1.20 LVU). This investigation revealed that enhanced bladder distension and increased bladder opacity, with a VBS surpassing 1.3 LVU, correlated with the presence of cystitis.

## Simple Summary

Urinary bladder inflammation, also known as cystitis, is a common and painful condition in pet guinea pigs that can markedly reduce their quality of life. Diagnosis is often challenging, and little information is available on the normal radiographic appearance of the guinea pig urinary bladder. In this study, we aimed to describe the size and appearance of the urinary bladder on radiographs in healthy guinea pigs and to compare these with those in guinea pigs showing clinical signs of cystitis. Radiographic images from 24 healthy animals were compared with those from 24 guinea pigs affected by cystitis. Guinea pigs with cystitis more frequently showed cloudy or mineral-like material within the urinary bladder and bladder stones. Their urinary bladders were also larger than those of healthy guinea pigs. To support an objective evaluation of urinary bladder size, we applied a simple measurement method based on the bones of the spine. The results show that radiographs can provide valuable information for identifying urinary bladder disease in guinea pigs and may help veterinarians diagnose cystitis more reliably, thereby improving animal welfare, but future larger studies are needed to confirm these results.

## 1. Introduction

In pet guinea pigs (*Cavia porcellus*), cystitis is one of the most common urinary tract diseases, with a propensity for recurrence that can severely affect their general well-being [1,2]. Older female guinea pigs are predisposed to developing cystitis and urinary tract infections [1,3,4]. Characteristic clinical signs of cystitis in guinea pigs include hematuria, incontinence, stranguria, and abdominal discomfort, often accompanied by inappetence and apathy [1]. A thorough diagnostic work-up is essential and includes a detailed medical history, clinical examination, abdominal radiography and ultrasonography, along with urinalysis and bacteriological urine culture [5,6].

Radiographic examination is essential for detecting urolithiasis [7], a frequent comorbidity in guinea pigs with urinary tract infections [1,3,8,9,10,11]. Martin [12] reported that 43% of guinea pigs presenting with clinical signs of urogenital tract disease suffered from urolithiasis, underscoring the diagnostic relevance of imaging in small mammals, particularly in cases of suspected urinary tract involvement.

Interpretation of the urinary bladder on radiographs is influenced by its variable filling status; in guinea pigs, the bladder is described as a pear-shaped organ situated within the pelvic cavity, with filling-related distension predominantly affecting its rounded cranial portion [13]. The bladder wall contains intramural ganglia, which play a functional role in the voiding cycle and continence, contributing to the guinea pig’s use as a model species in studies of bladder physiology [14,15]. Histomorphological studies have described the layered structure of the guinea pig bladder wall, including transitional epithelium, lamina propria, and smooth muscle bundles arranged in distinct orientations [13,16]. These anatomical features highlight the complexity of bladder function in guinea pigs. Despite such anatomical and histological insights, radiographic descriptions of the normal urinary bladder appearance in guinea pigs remain lacking. Furthermore, to date, no data have been published on the correlation between radiographic morphology of urinary bladders in guinea pigs and its traceability to clinically relevant diseases.

In contrast to dogs and cats, where cystitis typically presents without radiographic abnormalities [17], the potential for identifying such features in guinea pigs has not been investigated. This retrospective study was primarily conducted to assess the radiographic appearance of the urinary bladder in both healthy and cystitis-affected guinea pigs and to evaluate the feasibility of using radiographic imaging for objective measurement of bladder size.

## 2. Materials and Methods

### 2.1. Ethics Statement

The authors confirm adherence to the journal’s ethical policies as outlined in the author guidelines. This study involved a retrospective analysis of anonymized radiographic and clinical data obtained during routine diagnostic procedures in client-owned guinea pigs. No experimental interventions were performed, and no animals were specifically recruited or treated for research purposes. As such, ethical approval was not required.

### 2.2. Sample Selection

The database of AniCura Veterinary Clinic Haar GmbH was searched using the easyvet^©^ software (VetZ GmbH, Isernhagen, Germany, 2023) for guinea pigs in combination with various spellings and diagnostic terms associated with urinary tract infections. A retrospective review spanning 2009–2022 was conducted. Of 133 guinea pigs diagnosed with urinary tract infections, 24 met the inclusion criteria, which comprised urinalysis with evidence of leukocytes and/or bacterial infection diagnosed via cytology, alongside digital abdominal or whole-body radiographs and ultrasound and/or positive bacteriological urine culture. Radiographic selection criteria included precise patient positioning and clear visibility of the urinary bladder.

Exclusion criteria encompassed incomplete diagnostic work-up, poor radiographic quality, and suboptimal patient positioning that precluded consistent evaluation of the urinary bladder.

Data on age, sex, body weight, reason for presentation, comorbidities, causative organisms and their antimicrobial sensitivity, as well as therapeutic interventions were documented. Based on clinical history and recurrence patterns, the disease course was classified as chronic, defined by two or more distinct episodes of clinical cystitis within a 6-month period, or acute, characterized by complete clinical resolution and absence of recurrence throughout the follow-up period, as adapted from definitions used in feline and canine urinary tract infection studies [18,19]. The timing of radiographic imaging, whether at initial presentation or during the clinical course, was documented for each case.

A control group of 24 guinea pigs, matched by age with the cystitis group and possessing abdominal or whole-body radiographs, was selected. Inclusion criteria for the control group required that medical records indicated no specific clinical signs suggestive of urinary tract infection, as outlined by Azevedo et al. [1]. Animals in the control group were either clinically healthy at the time of presentation or diagnosed with conditions unrelated to the urinary tract (including disorders of the respiratory or gastrointestinal systems, musculoskeletal disease, or cardiac conditions).

### 2.3. Data Acquisition and Evaluation

Radiographic measurements, including urinary bladder height, vertebral bladder score (VBS), and marker-based assessments, were performed once by a single observer using a predefined measurement protocol. To minimize variability, the protocol was jointly reviewed and applied by both authors to a subset of radiographs, thereby standardizing the measurement approach before full analysis.

Digital radiographs, either whole-body or abdominal, were examined in lateral projection. Radiographs were obtained under routine clinical conditions, precluding standardized control of urinary bladder filling.

**Figure 1:**
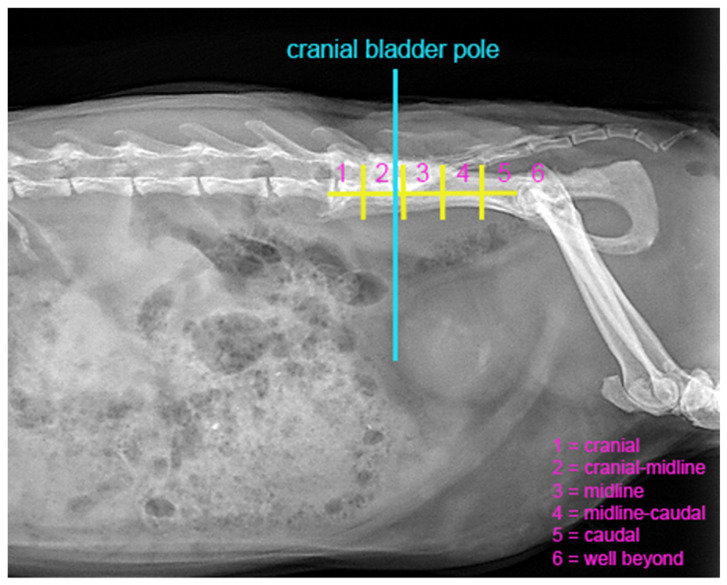
Radiograph of the abdomen of a guinea pig in right lateral projection, illustrating a mineral-dense shadow centrally located within the urinary bladder. The cranial bladder pole is defined by a vertical line extending from the cranial midpoint in the sagittal plane of the urinary bladder, which meets a straight line drawn from the iliac crest to the cranial acetabular rim. This line is divided into five equal segments, with a score of 6 indicating a position caudal to the fifth segment. The position of the vertical line in relation to the body of the ilium is classified as follows: 1: cranial; 2: cranial-midline; 3: midline; 4: midline-caudal; 5: caudal; 6: well beyond.

To objectively assess bladder distension, the position of the cranial bladder pole was determined in relation to the pelvis. First, the cranial bladder pole was marked by outlining a cranial midpoint in the sagittal plane of the bladder. A vertical line was then drawn upwards to the body of the ilium to precisely locate this reference point (Figure 1). To delineate the area of intersection, a straight line was drawn from the iliac crest to the cranial acetabular rim, subsequently divided into five equal segments. The position of the vertical line relative to those segments was recorded on a six-point scale: 1, cranial; 2, cranial-midline; 3, midline; 4, midline-caudal; 5, caudal; and 6, well beyond, referring to a position caudal to the fifth segment (Figure 1). Here, 1 indicated the greatest cranial extension of the bladder and thus the highest degree of distension, whereas 6 corresponded to minimal distension with the cranial bladder pole located furthest caudally.

**Figure 2:**
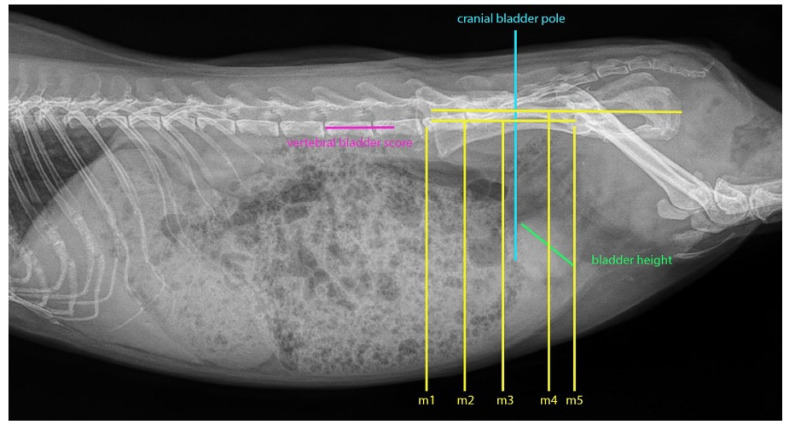
Radiograph of the abdomen of a guinea pig in right lateral projection. Marker lines, aligned with the bony landmarks of the pelvis, are employed to evaluate the precise location and extent of the urinary bladder by noting whether the cranial bladder pole reaches or surpasses these markers and identifying which marker is the most cranially positioned. Marker 1 (m1) represents the greatest bladder distension, while Marker 5 (m5) denotes the minimum distension. The cranial bladder pole is indicated by a vertical line extending from the cranial midpoint in the sagittal plane of the bladder upward toward the body of the ilium. Marker 1 (m1) is established as a vertical line from the cranial surface of the last lumbar vertebra. Marker 2 (m2) represents a vertical line from the caudal surface of the last lumbar vertebra. Marker 3 (m3) consists of a vertical line drawn from the iliac crest to the cranial edge of the acetabulum, with an additional perpendicular line extending centrally to the ventral abdominal wall. Marker 4 (m4) is formed by drawing a line from the iliac crest to the caudal end of the ischial tuberosity, passing through the centers of the femoral heads, from which a perpendicular line is derived. Marker 5 (m5) is illustrated as a diagonal line extending downward from the cranial edge of the acetabulum.

Furthermore, five marker lines were established to ascertain the exact location and size of the bladder (Figure 2). These lines were aligned with the pelvic bony landmarks and extended vertically down the abdominal wall. For each radiograph, observations were made regarding whether the cranial bladder pole extended to or beyond the markers, as well as identifying the marker it reached furthest cranially. Marker 1 represented the largest bladder, while marker 5 corresponded to the smallest.

Marker 1 was defined by a vertical line drawn from the cranial surface of the last lumbar vertebra. Marker 2 was positioned parallel to marker 1 but originated from the caudal surface of the last lumbar vertebra. Marker 3 was established by drawing a straight line from the iliac crest to the cranial acetabular rim and extending a perpendicular line from its midpoint to the ventral abdominal wall.

Marker 4 was generated from the iliac crest to the caudal end of the ischial tuberosity, with its height set at the midpoint of the femoral heads, and a perpendicular line drawn from its center. Marker 5 was indicated by a downward diagonal line from the cranial acetabular rim.

The presence of uroliths and/or mineral-dense shading (Figure 1) in the urinary bladder, including their distribution, was also documented.

To derive an objective measure of urinary bladder size, a parameter analogous to the vertebral heart score, termed the VBS, was employed. The maximum height of the urinary bladder was determined on right lateral radiographs using a millimeter ruler, measuring the distance between the ventral and dorsal surfaces along the transverse axis. This measurement was scaled against the length of the lumbar vertebrae, commencing from the cranial edge of the fifth lumbar vertebra and subsequently expressed in lumbar vertebral units (LVU) (Figure 2).

### 2.4. Statistical Analyses

For statistical analysis, Statistical Package for the Social Sciences (SPSS^®^ version 29.0; IBM Corp^©^, Armonk, NY, USA) was used. The VBS and measurements of the urinary bladder were tested for normal distribution, and accordingly, a *t*-test for independent samples or the Mann–Whitney U-test were performed to detect any significant differences in bladder distension between the control and cystitis groups. Categorical variables, including mineral-dense shading and solitary uroliths, were cross-tabulated and examined using the chi-square and Fisher’s exact test, informed by expected frequencies. A statistical significance threshold was established at *p* < 0.05.

## 3. Results

### 3.1. Signalment

The median age of the cystitis group was 4.4 years (range: 0.7–7 years), consisting of 20 intact females and 4 spayed females, with a median body weight of 0.95 kg (range: 0.67–1.32 kg). The control group comprised 19 intact females, 1 spayed female, and 4 neutered males, matched in age with the cystitis group, presenting a median weight of 1.05 kg (range: 0.73–1.3 kg).

### 3.2. Bacterial Isolates, Severity of Cystitis, and Timing of Radiographic Examinations

Bacteriological urine culture results (aerobic and anaerobic) were available for 15 guinea pigs. The most commonly isolated organism was *Corynebacterium renale* (*n* = 8). Other identified pathogens included a combination of *C. renale* and *Enterococcus* sp. (*n* = 1), *Staphylococcus* sp. (*n* = 2), *Escherichia coli* (*n* = 1), *Staphylococcus* sp. and *E. coli* co-isolated (*n* = 1), *Aerococcus viridans* (*n* = 1), and *Streptococcus* sp. (*n* = 1).

Within the cystitis cohort, 10 cases (42%) were classified as chronic and eight cases (33%) as acute. Euthanasia during the initial episode of disease was required in three guinea pigs (13%) due to severe cystitis and in one (4%) due to a concurrent comorbidity. One guinea pig (4%) died from severe cystitis, while another (4%) was lost to follow-up.

The evaluated radiographs were obtained at the time of initial presentation in 15 guinea pigs (62.5%) and at a later stage during the clinical course in nine animals (37.5%).

### 3.3. Urinary Bladder Size, Urolithiasis, and Mineral Density

The distribution of cranial bladder pole positions across both groups is summarized in Table 1. Fisher’s exact test revealed no statistically significant differences in cranial bladder pole position between the control and cystitis groups (*p* = 0.168).

Evidence of mineral-dense shading in the urinary bladder (Figure 1) was present in 11 patients (46%) of the cystitis group, with a predominance of centrally located shading (25%, 6/24). Solitary uroliths were identified in 25% of patients with cystitis, while the control group exhibited neither mineral-dense shading nor solitary uroliths. Fisher’s exact test showed a significant difference in the presence of uroliths (*p* two-sided = 0.022, *p* one-sided = 0.011). A comparable chi-square test for mineral density indicated a significant disparity (*p* ≤ 0.01) between patients with cystitis and the control group. Detailed frequencies are presented in Table 2.

The control group maximally achieved markers 3 and 4, with marker 5 being exceeded in all cases by both groups (Figure 2). In contrast, 25% of patients in the cystitis group reached markers 1 and 2. No significant difference in the maximum marker achieved was established (*p* = 0.092) using Fisher’s exact test, although two control group patients with tilted pelvic positions were excluded from the analysis.

### 3.4. Urinary Bladder Height and the VBS

**Figure 3:**
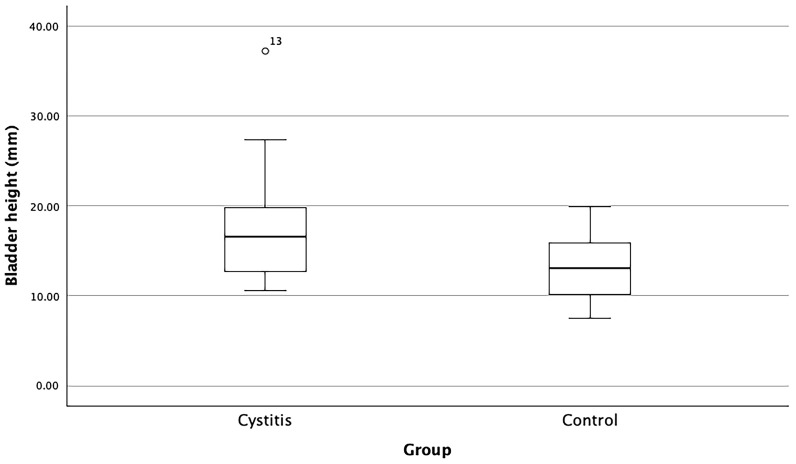
Box plots depicting the urinary bladder height of the cystitis group (*n* = 24) compared to the control group (*n* = 24) in right lateral view radiographs. This measurement is taken from the furthest point along the transverse axis of the bladder, representing the distance between the ventral and dorsal surfaces. The bladder height was significantly higher in the cystitis group than in the control group (*p* ≤ 0.01).

Bladder height was measured at the maximal transverse diameter, indicating the distance from ventral to dorsal surfaces. The median height in the cystitis group (16.6 mm, 95% confidence interval: 15.1–20.4 mm) was significantly greater than that observed in the control group (13.0 mm, 95% confidence interval: 11.6–14.6 mm) (Table 3, Figure 3), as confirmed by the Mann–Whitney U-test (*p* ≤ 0.01).

**Figure 4:**
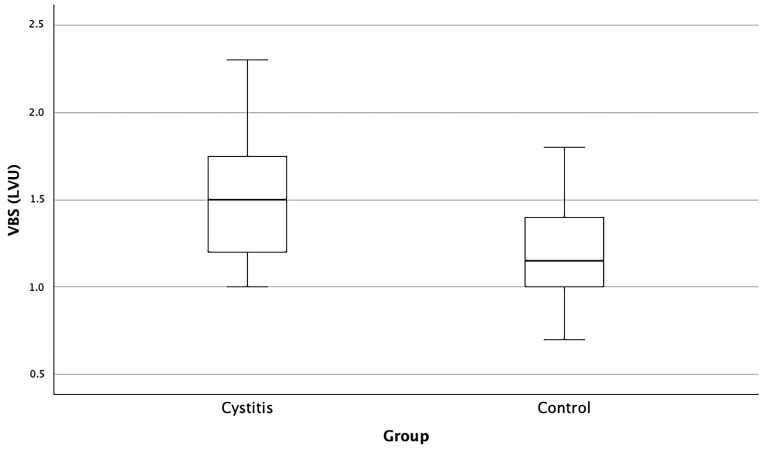
Box plots comparing the vertebral bladder scores (VBS) between the cystitis group (*n* = 24) and the control group (*n* = 24). The VBS represents the measurement of urinary bladder height projected to the cranial edge of the fifth lumbar vertebra, quantified as the number of vertebrae encompassed within the caliper points and expressed in lumbar vertebral units (LVU). The VBS was significantly higher in the cystitis group than in the control group (*p* (one-sided) = 0.003, *p* (two-sided) = 0.005).

The VBS exhibited significant variation between groups (*p* one-sided = 0.003, *p* two-sided = 0.005). The 95th percentile for the control group ranged from 1.1 to 1.3 LVU, whereas that for the cystitis group fell within 1.3 to 1.7 LVU. The median VBS for the cystitis group was 1.50 LVU, contrasting with a median of 1.2 LVU within the control cohort (Table 4, Figure 4).

## 4. Discussion

This study was conducted to evaluate the radiographic appearances of the urinary bladder in healthy guinea pigs compared to those of guinea pigs diagnosed with cystitis, as well as to introduce the VBS as a potential metric for bladder size assessment. Our findings revealed that cystitis significantly altered bladder opacity and size, with 46% of affected guinea pigs exhibiting increased opacity and a median bladder height of 16.6 mm compared to 13.0 mm in healthy controls. Moreover, the VBS was notably higher in patients with cystitis, indicating a discernible difference in bladder distension. These results not only enhance our understanding of the radiographic features associated with cystitis but also underscore the potential value of the VBS in clinical diagnostics.

Although urinary tract infection is a common disease in guinea pigs [20,21], consistent data are lacking, and no standardized protocol for diagnosis and treatment has been established. Nevertheless, it is widely agreed among practitioners that radiographic examination is indispensable in the clinical work-up to rule out urinary calculi, which often require surgical intervention [7,8]. Radiography is particularly suitable for this purpose due to the generally high mineral content of urinary calculi commonly detected in guinea pigs [9,22,23].

Mineral-dense shading in the bladder was identified in nearly half of the cystitis cases; however, it was absent in controls. This finding suggests that radiographic crystalluria may serve as a potential indicator of cystitis, although the conclusion is drawn from a limited sample size of 24 controls. Additionally, affected guinea pigs presented with bladder enlargement, in contrast to findings in dogs and cats, where pollakiuria typically correlates with reduced bladder size [17,24]. Stranguria and ventral urine soiling are commonly reported clinical signs in guinea pigs with cystitis [1], implying that pain might disrupt controlled bladder emptying, potentially by altering cholinergic neurotransmission [15]. This disruption could precipitate frequent passage of small amounts of urine and continuous dribbling, characteristic of overflow incontinence [15]. A less likely explanation is urine retention due to neurofunctional abnormalities. Most guinea pigs presenting with typical clinical signs of cystitis are not examined for neurological conditions, and no established methods exist to assess neurofunctional impairments of bladder function within this species. Although Maggi et al. [15] investigated neuroeffector mechanisms in the voiding cycle of the guinea pig bladder, research specifically addressing neurofunctional impairments and their clinical assessment remains lacking. In cats, however, neurofunctional abnormalities have been identified as the most relevant risk factor for developing urinary tract infections [25]. Therefore, analogous mechanisms warrant consideration in guinea pigs, as the causal relationship between neurological dysfunction and cystitis remains uncertain.

Reavill and Lennox [2] hypothesized that decreased urination frequency may facilitate bacterial proliferation and urolith formation, potentially stemming from physiological adaptations within guinea pigs, similar to chinchillas [26] and degus, to thrive in semi-arid environments with limited water resources [2]. In this context, an enlarged urinary bladder could be a predisposing factor for infection rather than a disease outcome. Moreover, urinary bladder size could be influenced by variables such as nutritional status. A documented correlation exists between body condition score and gastric distension in rabbits [27]. However, body weights in this study were nearly identical between groups, with the healthy control group presenting a marginally elevated median weight (50 g) over that of the cystitis group.

Several studies suggested a link between cystitis and urolithiasis in guinea pigs [1,3,8,9,10,11]. Our findings corroborate this literature, as none in the control group displayed urinary calculi, whereas one-quarter of those in the cystitis group did. Both conditions predominantly occur in middle-aged guinea pigs, and while urolithiasis has been reported more frequently in males in recent investigations [28], earlier studies described a slight predominance in females [8]. Notably, among guinea pigs diagnosed with concurrent bacterial cystitis and urolithiasis, no clear sex predisposition has been identified [9]. In addition, sex-specific differences in urolith localization have been described, with urethroliths occurring more commonly in females and cystoliths being more frequently observed in males [1]. The definitive causal relationship between urinary tract infections and urolithiasis remains to be determined. Damage to the bladder mucosa from urolith friction has been hypothesized to foster a conducive environment for bacterial colonization [3,29]. This is supported by the finding that *C. renale*, a common bacterial isolate in guinea pigs suffering from cystitis [8,9,10,30], preferentially colonizes damaged urogenital tissue [31]. However, urinary tract infections can also lead to the formation of urinary infection stones [32], as observed in rats [33].

Similar to the vertebral heart score used in guinea pigs [34], the VBS offers an objective metric accommodating variations in body size and mass, rendering it a convenient tool for clinical use. Marker 2 may serve as a practical tool owing to its swift application. In comparison to those with cystitis, our control group did not achieve this line, which may signify cystitis in cases where a significantly cranially extended bladder is present. However, nearly all animals reached markers 3, 4, and 5, indicating reduced specificity and, hence, limited clinical applicability. In routine clinical practice, the VBS may support a more objective assessment of bladder distension than visual estimation alone and may be useful for follow-up comparisons. However, interpretation remains influenced by organ overlap, patient positioning, and bladder filling status at the time of imaging. Therefore, the VBS and marker-based assessment should be considered complementary radiographic tools and not standalone diagnostic criteria.

This study has certain limitations. First, its retrospective design and relatively small sample size limit the generalizability of the findings, and the observed differences between groups may be susceptible to residual confounding. Therefore, the VBS and bladder height should be interpreted as supportive imaging parameters rather than as validated diagnostic thresholds. To establish reliable cut-off values, larger prospective studies are warranted.

Second, control group participants were selected based on medical history and the absence of specific clinical signs of urinary tract infection. Although these animals were deemed clinically healthy, recent urinalysis data were unavailable for all. This raises the possibility that subclinical urinary tract disease may have been present, which could have diminished the observed differences between groups. Future studies should ensure that urinalysis is performed systematically in all control animals to confirm the absence of urinary tract disease and strengthen the validity of group comparisons.

Third, nine guinea pigs within the cystitis group lacked bacteriological urine culture data due to limited owner compliance. These cases were included based on strong clinical suspicion for cystitis supported by diagnostic work-up and characteristic clinical signs. However, the absence of culture confirmation introduces diagnostic uncertainty and may have influenced case classification. Prospective investigations should ensure complete bacteriological confirmation to strengthen diagnostic accuracy.

Fourth, overlap in individual bladder height and VBS measurements was observed between groups. This suggests that single measurements alone have limited value for individual case classification. Nevertheless, increased values may provide an initial indication that further diagnostic evaluation for urinary tract disease is warranted. Future work should explore combining radiographic parameters with additional diagnostic modalities to improve case-level accuracy.

Fifth, radiographs were acquired under routine clinical conditions without standardized control of bladder filling. Bladder distension may therefore have been affected by the timing of imaging and individual voiding behavior. Consequently, selection bias may have been introduced, as the delineation of the bladder and precise positioning were selective criteria. Nonetheless, the impact of these criteria is likely minimal, given their consistent application across groups.

Finally, intra- and inter-observer agreements were not formally assessed; thus, measurement repeatability could not be quantified, which limits confidence in reproducibility. Although the measurement protocol was standardized and jointly reviewed prior to data acquisition, future prospective studies should include formal reliability testing to evaluate observer consistency.

## 5. Conclusions

This study provides preliminary insights into the radiographic characteristics of the urinary bladder in guinea pigs, highlighting the significant differences observed between healthy animals and those diagnosed with cystitis. The introduction of the VBS as a standardized measure for assessing bladder distension represents a promising approach for future diagnostic methodologies in small mammals. Our findings demonstrate that increased bladder opacity and size were indicators of cystitis, supporting the utility of radiographic imaging in clinical evaluations. However, given the retrospective design and relatively small sample size, these results should be interpreted with caution. Bladder size measurements and the VBS should be considered supportive imaging parameters rather than validated diagnostic thresholds. Larger, prospective studies are warranted to confirm clinically applicable cut-off values, further elucidate the pathophysiology of urinary tract diseases in guinea pigs, and enhance diagnostic accuracy in veterinary practice.

## Figures and Tables

**Table 1:** Cranial bladder pole position ^1^ on right lateral view radiographs of 24 healthy guinea pigs and 24 guinea pigs with clinical signs of cystitis.

Position		Control *n*%		Cystitis Group *n*%	
1	Cranial	2	8.3	3	12.5
2	Cranial-midline	5	20.8	10	41.7
3	Midline	11	45.8	11	45.8
4	Midline-caudal	3	12.5	0	0
5	Caudal	2	8.3	0	0
6	Well beyond	1	4.2	0	0
	Total	24	100	24	100

^1^ The cranial bladder pole position is defined by a vertical line from the cranial midpoint of the urinary bladder in the sagittal plane towards five equal segments from the iliac crest to the cranial acetabular rim. A score of 6 indicates a position beyond the fifth segment, with 1 representing the greatest and 6 representing the least.

**Table 2:** Occurrence of uroliths and mineral-dense shading in the urinary bladder on right lateral view radiographs of 24 healthy guinea pigs and 24 guinea pigs with clinical signs of cystitis.

		Control *n*%		Cystitis Group *n*%	
Mineral-dense shading	Present	0	0	11	45.8
	Not present	24	100	13	54.2
	Total	24	100	24	100
Uroliths	Present	0	0	6	25
	Not present	24	100	18	75
	Total	24	100	24	100

**Table 3:** Descriptive statistics of urinary bladder height ^1^ on right lateral view radiographs of 24 healthy guinea pigs and 24 guinea pigs with clinical signs of cystitis.

Parameter	Control	Cystitis Group
Median (mm)	13.0	16.6
Minimum (mm)	7.5	10.6
Maximum (mm)	19.9	37.2
Mean (mm)	13.1	17.7
Standard deviation (mm)	3.6	6.3

^1^ Urinary bladder height is measured along a line at the furthest point of the transverse bladder axis, which is equivalent to the distance between the ventral and dorsal surfaces.

**Table 4:** Descriptive statistics of the vertebral bladder score ^1^ (expressed in lumbar vertebral units, LVU) on right lateral view radiographs of 24 healthy guinea pigs and 24 guinea pigs with clinical signs of cystitis.

Parameter	Control	Cystitis Group
Median (LVU)	1.2	1.5
Minimum (LVU)	0.7	1.0
Maximum (LVU)	1.8	2.3
Mean (LVU)	1.2	1.5
Standard deviation (LVU)	0.3	0.4

^1^ The vertebral bladder score refers to the urinary bladder height projected to the cranial edge of the fifth lumbar vertebra, measured as the number of vertebral bodies encompassed within the caliper points (LVU).

## Data Availability

The data that support the findings of this study are available from the corresponding author upon reasonable request.

## Abbreviations

The following abbreviations are used in this manuscript:

VBS	Vertebral bladder score
LVU	Lumbar vertebral units
